# Supplementary material for: EvoAug: improving generalization and interpretability of genomic deep neural networks with evolution-inspired data augmentations
Source: Genome Biol. 2023 May 5;24:105. doi: 10.1186/s13059-023-02941-w (PMC10161416; doi:10.1186/s13059-023-02941-w)
Supplement: Supplementary file 1 — Additional file 1. Supplementary Tables S1-S3 and Figures S1-S7. [file 13059_2023_2941_MOESM1_ESM.pdf]

Table S1: ENCODE ChIP-seq details. Ten representative TF ChIP-seq experiments in GM12878 cell line and a DNase-seq experiment (File accession: ENCFF235KUD) for the same cell line were downloaded from ENCODE. Table shows ENCODE file accession codes for all transcription factor proteins.

PROTEIN	ENCODE FILE ACCESSION	CELL LINE
MAX	ENCFF083KVY	GM12878
BACH1	ENCFF012JXJ	GM12878
GABPA	ENCFF116EXQ	GM12878
ZNF24	ENCFF103OOV	GM12878
ELK1	ENCFF556JBS	GM12878
SRF	ENCFF909FRA	GM12878
REST	ENCFF677KJB	GM12878
ATF2	ENCFF127GYQ	GM12878
CTCF	ENCFF710VEH	GM12878

Table S2: Computational cost for Basset models. Table shows the computational cost of EvoAug augmentations for Basset models, including the number of epochs, time per epoch, and the total training time, on a single NVIDIA RTX 2080ti GPU. The values represent the average across 5 independent trials and errors represent standard deviation of the mean.

AUGMENTATION	EPOCHS	TIME PER EPOCH (S)	TOTAL TIME (MIN)
STANDARD	14.0 ± 1.3	74	17.3 ± 1.6
NOISE	35.4 ± 9.7	98	57.8 ± 15.8
RC	25.4 ± 5.8	114	48.3 ± 11.0
MUTATION	52.4 ± 12.5	171	149.3 ± 35.625
TRANSLOCATION	25.6 ± 3.3	125	53.3 ± 6.9
DELETION	32.2 ± 6.1	172	92.3 ± 17.5
INSERTION	34.0 ± 8.3	174	98.6 ± 24.1
NOISE+RC+INS	87.6 ± 12.2	219	319.7 ± 44.5
ALL	85.4 ± 15.1	259	368.6 ± 65.2

Table S3: Computational cost for DeepSTARR models. Table shows the computational cost of EvoAug augmentations for DeepSTARR models, including the number of epochs, time per epoch, and the total training time, on on a single NVIDIA RTX 2080ti GPU. The values represent the average across 5 independent trials and errors represent standard deviation of the mean.

Augmentation	Epochs	Time per epoch (s)	Total time (min)
Standard	22.4 ± 1.4	62	23.1 ± 1.4
Noise	45.0 ± 11.1	76	57.0 ± 14.1
Mutation	49.6 ± 8.0	138	114.1 ± 18.4
Translocation	58.6 ± 12.4	108	105.5.1 ± 22.3
Deletion	56.6 ± 11.2	149	140.6 ± 27.8
Insertion	52.8 ± 13.6	156	137.3 ± 35.4
Noise+Ins	81.8 ± 16.0	217	295.8 ± 57.9
All	74.4 ± 10.0	264	327.4 ± 44.0

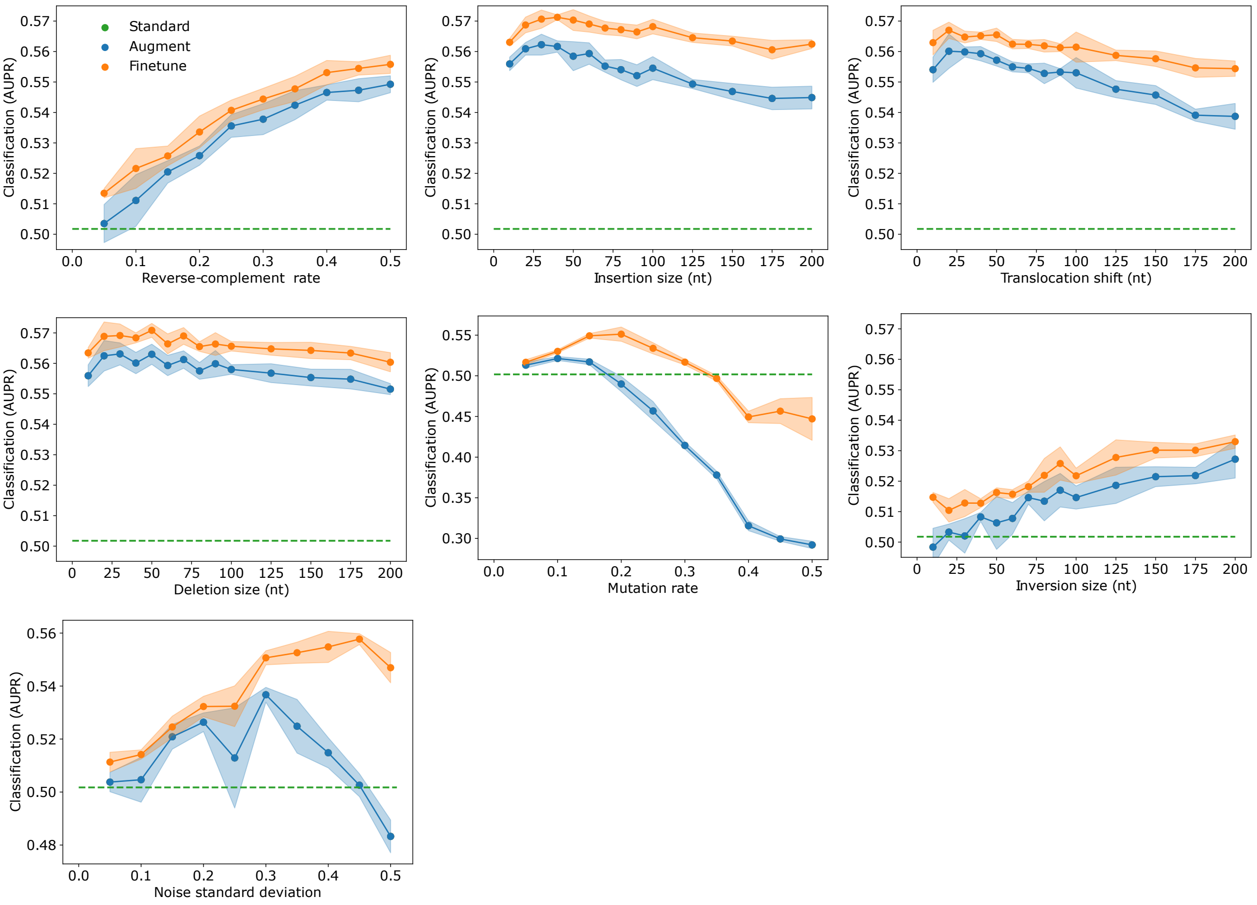

Fig S1: Hyperparameter sweep of each augmentation method for Basset. Each plot shows the average classification performance for different hyperparameter values intrinsic to each data augmentation method. Shaded region represents the standard deviation of the mean. Dashed line represents the performance without augmentations. Values reported are with $n = 5$ trials with random initializations.

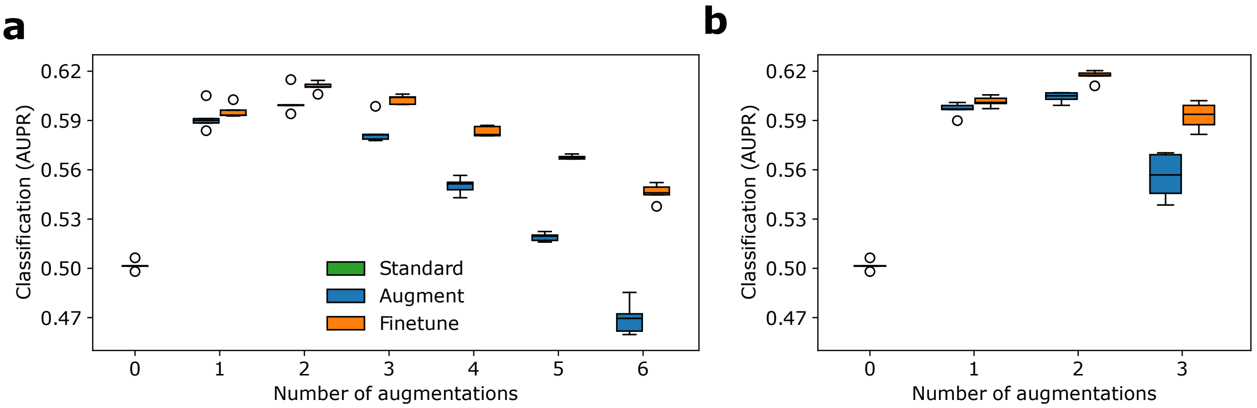

Fig S2: Sweep in number of applied augmentations for Basset. (**a**) Box-plot of classification performance for Basset models pre-trained with two combination strategies of (**a**) all and (**b**) insertion + translocation + deletion, and fine-tuned on original data. All represents reverse-complement, Gaussian noise, insertion, deletion, translocation, and mutation. Standard represents no augmentations during training. Each number in the x-axis represents the number of augmentations that are applied to each sequence in combinations during training. Box plots show the first and third quartiles, central line is the median, and the whiskers show the range of data with outliers removed. Values reported are with $n = 5$ trials with random initializations.

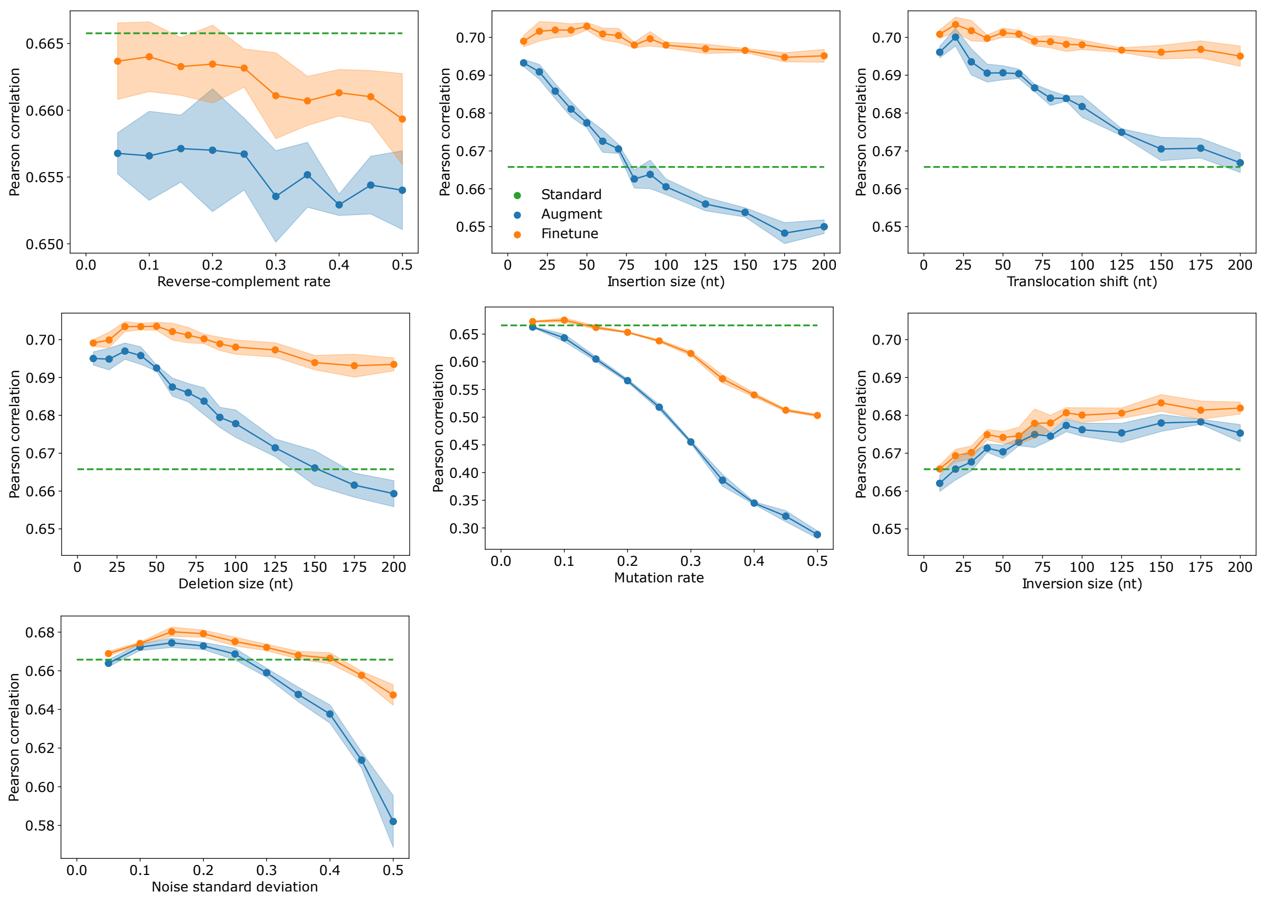

Fig S3: Hyperparameter sweep of each augmentation method for DeepSTARR, Developmental. Each plot shows the average classification performance for different hyperparameter values intrinsic to each data augmentation method. Shaded region represents the standard deviation of the mean. Dashed line represents the performance without augmentations. Values reported are with $n = 5$ trials with random initializations.

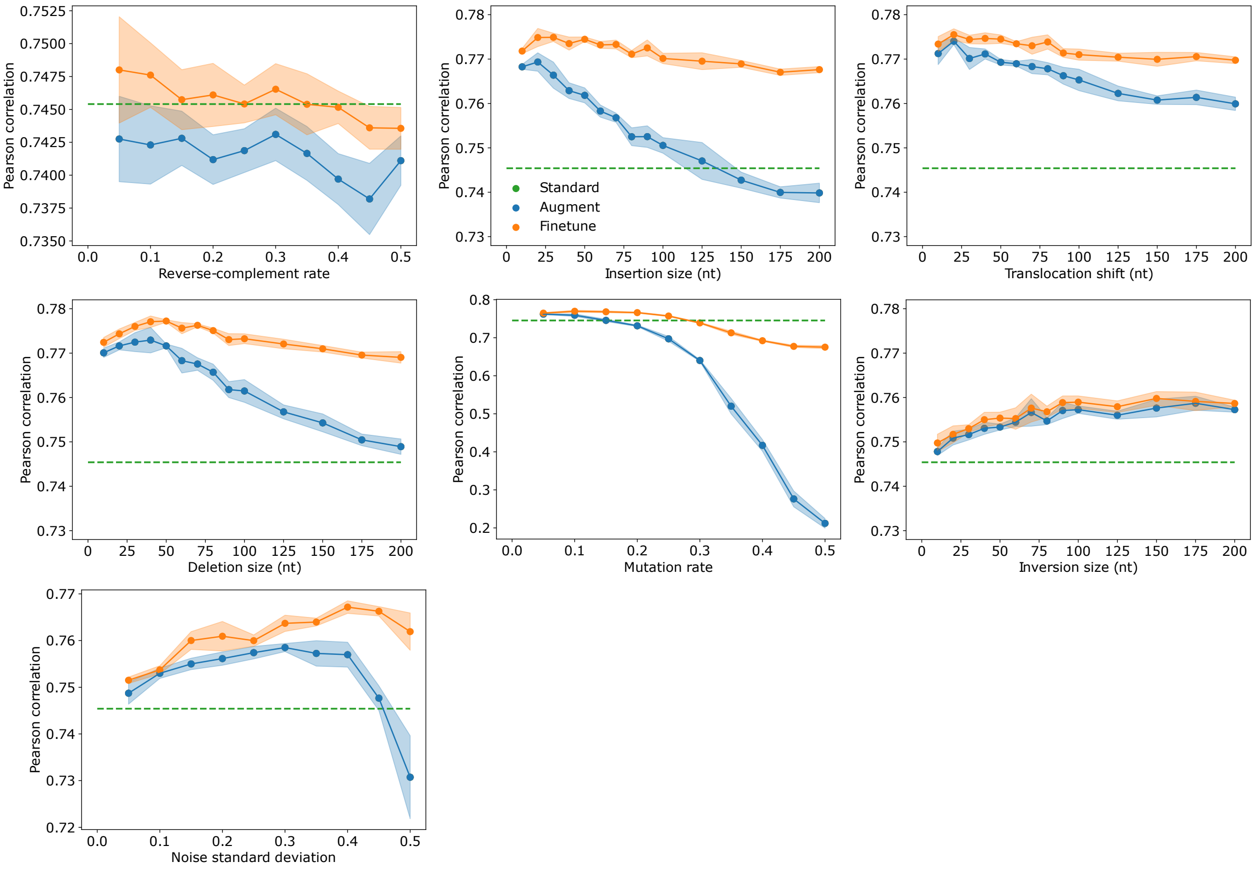

Fig S4: Hyperparameter sweep of each augmentation method for DeepSTARR, Housekeeping. Each plot shows the average classification performance for different hyperparameter values intrinsic to each data augmentation method. Shaded region represents the standard deviation of the mean. Dashed line represents the performance without augmentations. Values reported are with $n = 5$ trials with random initializations.

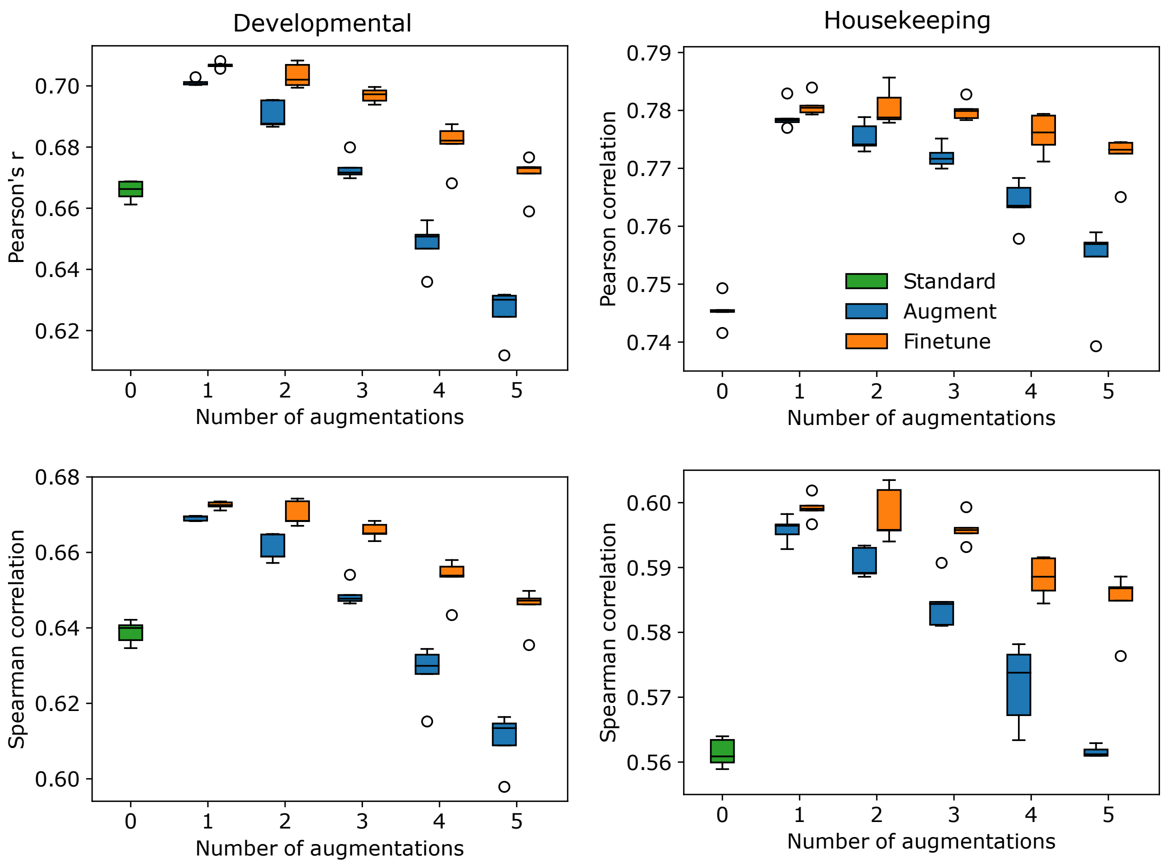

Fig S5: Sweep in number of applied augmentations for DeepSTARR. Box-plot of regression performance (Pearson's r for top row and Spearman correlation for bottom row) for DeepSTARR models pre-trained with all augmentations—namely, reverse-complement, Gaussian noise, insertion, deletion, translocation, and mutation—for developmental (left) and housekeeping (right) conditions. Standard represents no augmentations during training. Each number in the x-axis represents the number of augmentations that are applied to each sequence in combinations during training. Box plots show the first and third quartiles, central line is the median, and the whiskers show the range of data with outliers removed. Values reported are with $n = 5$ trials with random initializations.

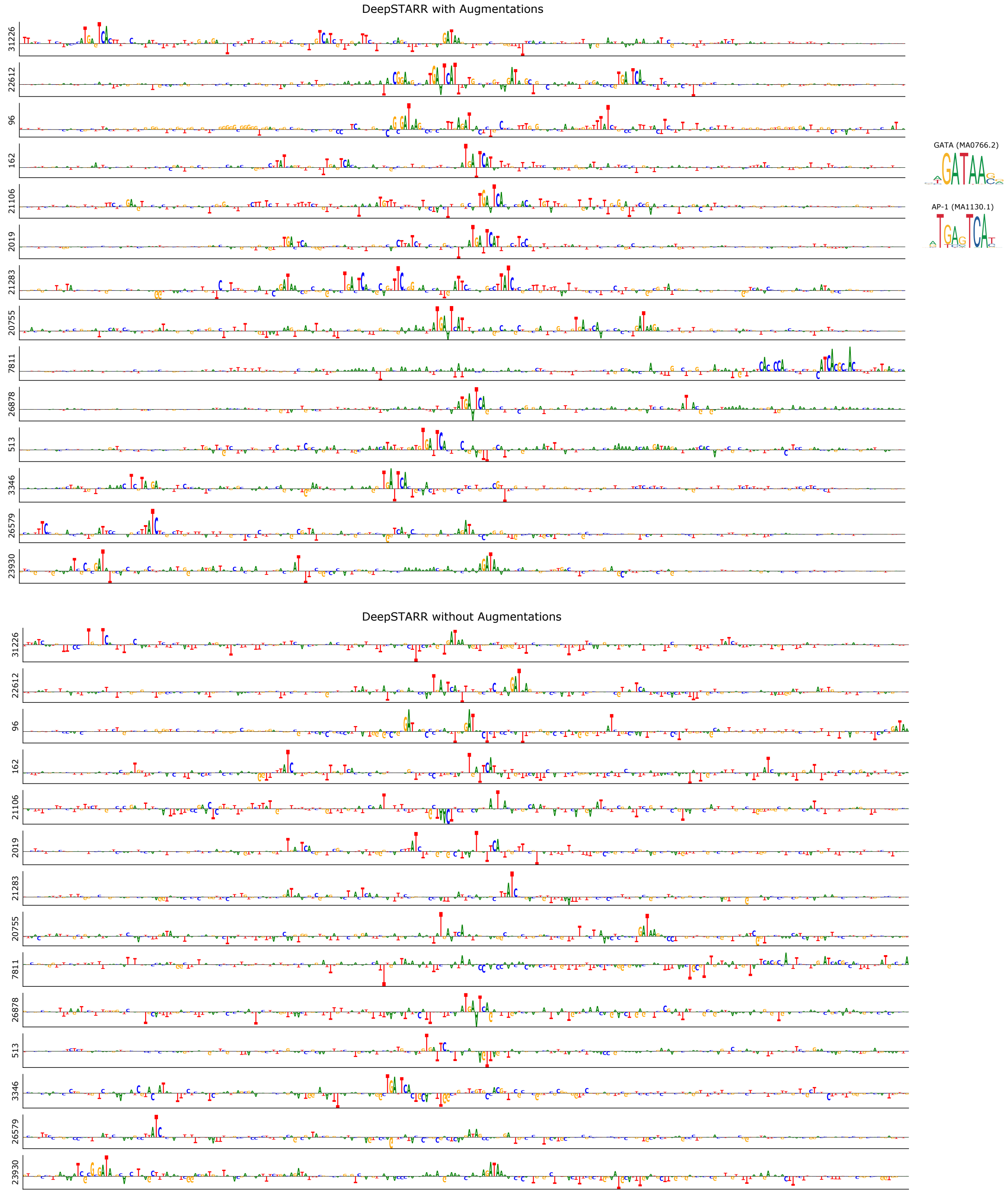

Fig S6: Attribution map comparison for DeepSTARR. Sequence logos of SHAP-based attribution maps for a fine-tuned DeepSTARR model that was pretrained with a combination of all augmentations up to 2 augmentations total per sequence (top) and DeepSTARR trained without any augmentations (bottom). The number on the *y*-axis represents the index of the sequence from the DeepSTARR test set. Sequence logos of GATA and AP1 are shown for visual comparison.

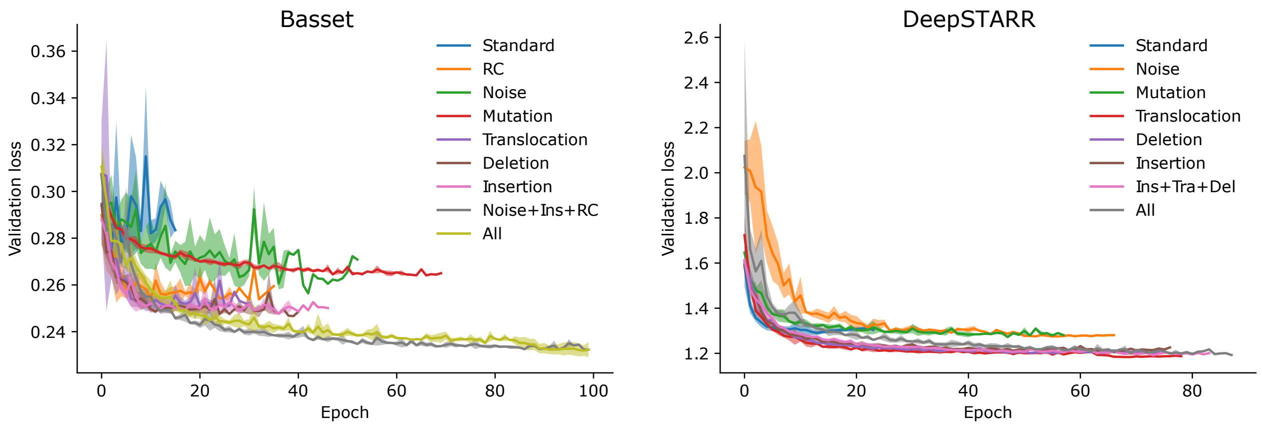

Fig S7: Training performance. The average validation loss at each epoch of training for various augmentations applied to Basset (left) and DeepSTARR (right). Values reported are with $n = 5$ trials with random initializations. Shaded region represents the standard deviation. Due to the different training times for each trial, standard deviation was calculated for epochs that contained at least 3 values.
