# Supplementary material for: EvoAug: improving generalization and interpretability of genomic deep neural networks with evolution-inspired data augmentations
Source: Genome Biol. 2023 May 5;24:105. doi: 10.1186/s13059-023-02941-w (PMC10161416; doi:10.1186/s13059-023-02941-w)
Supplement: Supplementary file 2 — Additional file 2. Review history. [file 13059_2023_2941_MOESM2_ESM.docx]

**1^st^ round**

**Reviewer 1**

In this short manuscript, the authors present a data augmentation approach for learning DNA-input deep neural network (DNN) models. Data augmentation is an approach for creating extra training data for training a machine learning model by altering the existing training data. The authors propose augmentations that make seven types of modifications to the input sequences--including single-nucleotide mutation, insertion and deletion--while leaving the labels the same. They evaluate this approach on the Basset task of predicting DNase-seq data. They find that training using augmentations improves performance according to several measures: (1) prediction accuracy on held-out examples; (2) accuracy at predicting the effects of saturation mutagenesis as measured by massively parallel reporter assays, a task defined by the CAGI5 challenge; (3) how well convolutional filters match known transcription factor PWMs, and (4) prediction of Drosophila STARR-seq data. The authors find that augmentations tend to improve performance for tasks 1,2 and 4. They tend to decrease or leave the same performance on task 3. These results are a bit surprising because there is no guarantee that the proposed augmentations leave DNase-seq unchanged (although this problem is mitigated by a fine-tuning step without augmentation).

Overall, the manuscript presents a nice improvement that can be applied across DNA-input DNN models. The text and figures are clear. I have a few relatively minor comments.

Comments:

It is hard to put the improvement in accuracy in context. This could be done easily in the case of CAGI5: what was the distribution of scores for the top-scoring teams?

What is the computational cost of adding augmentations? I would imagine that the time for each epoch is not significantly changed, but that early stopping is reached less often because there are effectively more data points. Also, another approach for gaining increased accuracy at the expense of computation would be to train across more data types (TF and histone ChIP-seq, CAGE, etc) as is done by recent methods like Enformer. This should be at least mentioned.

Minor notes:

Fig 1a "pre-training": This is a bit confusing because the same amount of data is shown before and after augmentation. I understand that augmentation doesn't change the size of each mini-batch, but it simulates drawing examples from a larger training set.

"Gaussian noise: a transformation where Gaussian noise (with distribution parameters noise mean = 0 and noise std) is added to the input sequence." Is this Gaussian noise added to the 1-hot encoded vectors representing DNA sequence?

It would be valuable to include the plot of generalization performance as a function of epochs in the supplement. If augmented data performs better even in the first epoch, this would suggest that something about the noise introduced by augmentation helps training even when it replaces real data.

A caveat that should be mentioned: Data augmentation provides a bias-variance tradeoff. Data augmentation reduces variance by increasing the size of the training data. It increases bias because the assumption that the true label is invariant after perturbation may not always be correct. As the availability of training data increases (such as by training on more data types), augmentation should become less relevant. That said, the Basset data set is already quite large.

**Reviewer 2**

The work evaluate deep learning models' argument the deep learning model using evolutionary information in the genomic sequence such as insertion/deletion, translation to demonstrate that the augmented deep learning model will outperform the non-argumented version in different context. The work select two popular deep learning models DeepSTARR and BASSET to evaluate the argumentation approach. I have some major comments as follows


1. One comment is about the intuition to develop the argumentation using genomic mutations in the sequence. Though the sample size will increase by including both reference genome and mutated genome, most of the sequence information will remain the same (e.g. insertion and deletion are usually very small). Thus, the augmented samples are duplicated in most cases and seems not contributed much in the prediction intuitively.

2. A relevant comment is using genomic sequence for prediction, in most cases, the sample size is large enough (e.g. BASSET) for training a robust deep learning model. Further increasing the sample size will not helpful to improve the model further.

3. The evaluated deep learning models for the augmentation approach are limited to two. Many more deep learning models are available for genomic prediction task such as DeepSEA, DanQ, etc.

**Reviewer 3**

In this short report format article, authors N. K. Lee and colleagues present data augmentation techniques applied to genomics datasets to train deep neural networks that enable the model to generalize better to unseen data compared to the standard non-augmented genomics datasets in the context of regulatory genomics prediction tasks such as the prediction of transcription factor binding sites, sites of chromatin accessibility, enhancer activities, and impact of cis-regulatory region mutations.

Data augmentation techniques have been proven useful in training more generalizable models in the computer vision field such as for image classification tasks, where an image can be cropped, rotated, transformed, modified so that the model learns the relevant features for the actual content of the image rather than the spurious features that lead the model to overfit to the current set of observations. For image analysis, there exist many such data augmentations methods, however, such a variety is currently missing in omics data science. The importance of the availability of data augmentation techniques is even more pronounced for omics data analysis, because the number of samples available in an experiment are usually on the order of tens/hundreds. Motivated by these points, the authors tackle the problem of generating synthetic genomic data from a given genomic dataset by creating random small/large mutations in the input sequences. One major bottleneck for data augmentation for genomic data is that, in contrast to the image analysis, where a cropped/rotated/transformed/filtered image still preserves its label, a mutated genomic sequence might not necessarily still have the same functionality as the wild type version. The authors apply a smart and simple trick to overcome this and demonstrate that a model first trained on augmented dataset and finetuned on the original dataset can not only generalize better to holdout data, but also shows robust performance in terms of the chosen hyperparameters which determine the level of augmentation applied on the original dataset.

The manuscript is overall well written (except for a few minor points, see below), the figures are well illustrated. I can't see any issues with the methodology, the overall machine learning workflow is properly followed. The code, which also seems to be clearly written, is publicly available. I support the publication of this paper in its current form. I expect to see more and more such approaches applied to not only genomic data, but also other layers of the omics profiling such as the transcriptome, proteome, and the epigenome.

I have some minor comments to improve the text and optional suggestions that could be implemented as part of this work (if time permits) or in future versions of the software.

- Probably due to the word count limits of the short report format, the text was sometimes too compactly written. A little more explanation could help in certain parts. For instance, a summary of the prediction tasks that have been studied could be added before starting the results section.
- Again probably due to the word limit, a discussion of future directions is missing. What other tasks can this method be used on? Can one use these data augmentations to predict for instance the effect of genomic changes on the target gene expression levels (as in GTEX)? It would be nice to see a few examples discussed in the discussion sections to see how else this tool could be used.
- The authors have demonstrated the generalization power of their training procedure on various tasks, but all of them are within-dataset context. It would be an open question how the model would generalize in a cross-dataset context where the distributions could be more shifted. Possibly a second fine-tuning to the external dataset could solve this issue. Maybe the authors could comment on this in the discussion section.
- Data augmentation brings up the idea of using contrastive learning approaches using for instance contrastive/triplet loss, which can also be used in a supervised setting. I wonder if the authors considered following such an approach and if it makes any difference.
- The authors apply two different sets of hyperparameter optimization: one for learning the neural network model training parameters and one for learning the data augmentation parameters (such as mutation rate or length and combinations of different kinds of augmentations). Hyperparameters are randomly generated on a grid and best performing parameters are selected. This sounds like a good use case where a population based training could be used to detect optimum hyperparameters concurrently for both the augmentation parameters and the model training parameters (See https://arxiv.org/pdf/1905.05393.pdf). In such a setting, the algorithm would actually evolve a set of solutions on the go. As the intermediate solutions that perform well are not discarded but exploited for the subsequent solutions while at the same time being mutated, converging on the best fitting hyperparameter setup could be more efficient for population based training compared to random grid search. This can be done using tools such as Ray Tune that can easily be connected to Pytorch code.

**Authors’ response**

Reviewer #1: In this short manuscript, the authors present a data augmentation approach for learning DNA-input deep neural network (DNN) models. Data augmentation is an approach for creating extra training data for training a machine learning model by altering the existing training data. The authors propose augmentations that make seven types of modifications to the input sequences--including single-nucleotide mutation, insertion and deletion--while leaving the labels the same. They evaluate this approach on the Basset task of predicting DNase-seq data. They find that training using augmentations improves performance according to several measures: (1) prediction accuracy on held-out examples; (2) accuracy at predicting the effects of saturation mutagenesis as measured by massively parallel reporter assays, a task defined by the CAGI5 challenge; (3) how well convolutional filters match known transcription factor PWMs, and (4) prediction of Drosophila STARR-seq data. The authors find that augmentations tend to improve performance for tasks 1,2 and 4. They tend to decrease or leave the same performance on task 3. These results are a bit surprising because there is no guarantee that the proposed augmentations leave DNase-seq unchanged (although this problem is mitigated by a fine-tuning step without augmentation).

Overall, the manuscript presents a nice improvement that can be applied across DNA-input DNN models. The text and figures are clear. I have a few relatively minor comments.

Comments:

It is hard to put the improvement in accuracy in context. This could be done easily in the case of CAGI5: what was the distribution of scores for the top-scoring teams?

In the revised manuscript, we provide additional context for how well these scores compare against other deep learning models and top-scoring teams on the CAGI5 challenge.

What is the computational cost of adding augmentations? I would imagine that the time for each epoch is not significantly changed, but that early stopping is reached less often because there are effectively more data points. Also, another approach for gaining increased accuracy at the expense of computation would be to train across more data types (TF and histone ChIP-seq, CAGE, etc) as is done by recent methods like Enformer. This should be at least mentioned.

Great point. Data augmentations indeed introduce an added computational cost. In the revised manuscript, we have added a brief discussion about this computational cost, including 2 additional tables and a supplemental figure that shows training curves. As shown in Supplementary Tables 2 and 3, the time for each epoch does vary depending on the specific augmentations used, and we also observed that it took longer for models to reach convergence when using EvoAug compared to when no augmentations were used. However, we did find that the training curves appeared to be more stable for the Basset dataset (Supplementary Fig. 7). This is certainly an advantage as the convergence to better generalizing models is consistent, despite the computational overhead.

Minor notes:

Fig 1a "pre-training": This is a bit confusing because the same amount of data is shown before and after augmentation. I understand that augmentation doesn't change the size of each mini- batch, but it simulates drawing examples from a larger training set.

While the underlying dataset is the same, the training process in stage 1 of EvoAug does involve an "augmented" versions of the data. In the revised manuscript, we have clarified the terminology by referring to the two stages of training as "stage 1" and "stage 2" of a training curriculum, instead of using the term "pretraining." This should help mitigate any confusion with pretraining techniques that use large amounts of data.

"Gaussian noise: a transformation where Gaussian noise (with distribution parameters noise mean = 0 and noise std) is added to the input sequence." Is this Gaussian noise added to the 1- hot encoded vectors representing DNA sequence?

This is accurate. The EvoAug method adds a different Gaussian noise to each element in the one-hot

encoded DNA sequence, which samples outside of the simplex where the data resides. As a result, maintaining the same label encourages the model to learn a flat function off the simplex. This property could have an added benefit that smoothens the learned function, making the model more amenable to gradient-based attribution methods.

It would be valuable to include the plot of generalization performance as a function of epochs in the supplement. If augmented data performs better even in the first epoch, this would suggest that something about the noise introduced by augmentation helps training even when it replaces real data.

Agreed. In the revised manuscript, we have included this plot in supplementary figure 7. We have also added additional discussion to describe the benefits of a more stable training, as observed in reduced fluctuations in the validation loss.

A caveat that should be mentioned: Data augmentation provides a bias-variance tradeoff. Data augmentation reduces variance by increasing the size of the training data. It increases bias because the assumption that the true label is invariant after perturbation may not always be correct. As the availability of training data increases (such as by training on more data types), augmentation should become less relevant. That said, the Basset data set is already quite large.

We thank the reviewer for bringing up this important point. In response, we have added a more detailed discussion in the revised manuscript. We do acknowledge that as the data size increases, the effectiveness of data augmentations is expected to decrease. However, the extent to which this occurs depends on the complexity of the features in the data. For example, in the case of the Basset dataset, which is quite large, EvoAug provides significant performance improvements, suggesting that augmentations can still be beneficial even with large datasets. On the other hand, simpler datasets, such as chip-seq, may not observe as much gains as the data size increases. This was demonstrated in the ChIP-seq analysis (Fig. 2). We appreciate the reviewer's feedback and hope that our revised discussion provides some considerations on the relationship between data size, complexity, and the effectiveness of data augmentations.

Reviewer #2: The work evaluate deep learning models' argument the deep learning model using evolutionary information in the genomic sequence such as insertion/deletion, translation to demonstrate that the augmented deep learning model will outperform the non-argumented version in different context. The work select two popular deep learning models DeepSTARR and BASSET to evaluate the argumentation approach. I have some major comments as follows

1. One comment is about the intuition to develop the argumentation using genomic mutations in the sequence. Though the sample size will increase by including both reference genome and mutated genome, most of the sequence information will remain the same (e.g. insertion and deletion are usually very small). Thus, the augmented samples are duplicated in most cases and seems not contributed much in the prediction intuitively.

Ideally, data augmentations in genomics would add genetic variation in non-critical regions of the sequence, providing additional information to the model about what is important and what is not. This is similar to placing a foreground object in different backgrounds in computer vision. Although the central information (i.e., foreground object) remains the same, showing variability in the background provides additional information (i.e., bias) that the background is not as important. However, implementing this in practice for genomics is challenging. Therefore, we chose to impose augmentations that could alter the function of the modified sequence, but we address this issue through a 2-stage training curriculum. In the revised manuscript, we provide additional clarifying text to emphasize the benefits of genetic variation in non-critical regions of the sequence.

2. A relevant comment is using genomic sequence for prediction, in most cases, the sample size is large enough (e.g. BASSET) for training a robust deep learning model. Further increasing the sample size will not helpful to improve the model further.

While this hypothesis seems reasonable, our practical results show that augmenting already large datasets like Basset further improves model performance, contradicting this assumption. We believe that the benefits of augmentation depend not only on the dataset size, but also on the complexity of the underlying features to be learned. For example, the chip-seq datasets with a larger amount of data only showed a slight improvement compared to the chip-seq datasets with less data (Fig. 2).

3. The evaluated deep learning models for the augmentation approach are limited to two. Many more deep learning models are available for genomic prediction task such as DeepSEA, DanQ, etc.

DeepSea and DanQ analyzed a more comprehensive version of the Basset dataset, including histone marks and TF binding obtained from chip-seq experiments in ENCODE. Our study, on the other hand, focused on demonstrating the efficacy of data augmentations on two multi-task datasets and nine single-task datasets, for a total of 11 datasets. While there are many other datasets that we could have tested on, we chose to concentrate on representative computational genomics prediction tasks to showcase the generalization of our approach across different types of prominent prediction tasks. We have revised the manuscript to reflect this distinction more accurately.

Supplementary Table 2. Computational cost for Basset models. Table shows the computational cost of EvoAug augmentations for Basset models, including the number of epochs, time per epoch, and the total training time, on a single NVIDIA RTX 2080ti GPU. The values represent the average across 5 independent trials and errors represent standard deviation of the mean.

AUGMENTATION EPOCHS TIME PER EPOCH (S) TOTAL TIME (MIN)

STANDARD 14.0 ± 1.3 74 17.3 ± 1.6

NOISE 35.4 ± 9.7 98 57.8 ± 15.8

RC 25.4 ± 5.8 114 48.3 ± 11.0

MUTATION 52.4 ± 12.5 171 149.3 ± 35.625

TRANSLOCATION 25.6 ± 3.3 125 53.3 ± 6.9

DELETION 32.2 ± 6.1 172 92.3 ± 17.5

INSERTION 34.0 ± 8.3 174 98.6 ± 24.1

NOISE+RC+INS 87.6 ± 12.2 219 319.7 ± 44.5

ALL 85.4 ± 15.1 259 368.6 ± 65.2

Supplementary Table 3. Computational cost for DeepSTARR models. Table shows the computational cost of EvoAug augmentations for DeepSTARR models, including the number of epochs, time per epoch, and the total training time, on on a single NVIDIA RTX 2080ti GPU. The values represent the average across 5 independent trials and errors represent standard deviation of the mean.

AUGMENTATION EPOCHS TIME PER EPOCH (S) TOTAL TIME (MIN)

STANDARD 22.4 ± 1.4 62 23.1 ± 1.4

NOISE 45.0 ± 11.1 76 57.0 ± 14.1

MUTATION 49.6 ± 8.0 138 114.1 ± 18.4

TRANSLOCATION 58.6 ± 12.4 108 105.5.1 ± 22.3

DELETION 56.6 ± 11.2 149 140.6 ± 27.8

INSERTION 52.8 ± 13.6 156 137.3 ± 35.4

NOISE+INS 81.8 ± 16.0 217 295.8 ± 57.9

ALL 74.4 ± 10.0 264 327.4 ± 44.0

Reviewer #3: In this short report format article, authors N. K. Lee and colleagues present data augmentation techniques applied to genomics datasets to train deep neural networks that enable the model to generalize better to unseen data compared to the standard non- augmented genomics datasets in the context of regulatory genomics prediction tasks such as the prediction of transcription factor binding sites, sites of chromatin accessibility, enhancer activities, and impact of cis-regulatory region mutations. Data augmentation techniques have been proven useful in training more generalizable models in the computer vision field such as for image classification tasks, where an image can be cropped, rotated, transformed, modified so that the model learns the relevant features for the actual content of the image rather than the spurious features that lead the model to overfit to the current set of observations. For image analysis, there exist many such data augmentations methods, however, such a variety is currently missing in omics data science. The importance of the availability of data augmentation techniques is even more pronounced for omics data analysis, because the number of samples available in an experiment are usually on the order of tens/hundreds. Motivated by these points, the authors tackle the problem of generating synthetic genomic data from a given genomic dataset by creating random small/large mutations in the input sequences. One major bottleneck for data augmentation for genomic data is that, in contrast to the image analysis, where a cropped/rotated/transformed/filtered image still preserves its label, a mutated genomic sequence might not necessarily still have the same functionality as the wild type version. The authors apply a smart and simple trick to overcome this and demonstrate that a model first trained on augmented dataset and finetuned on the original dataset can not only generalize better to holdout data, but also shows robust performance in terms of the chosen hyperparameters which determine the level of augmentation applied on the original dataset.

The manuscript is overall well written (except for a few minor points, see below), the figures are well illustrated. I can't see any issues with the methodology, the overall machine learning workflow is properly followed. The code, which also seems to be clearly written, is publicly available. I support the publication of this paper in its current form. I expect to see more and more such approaches applied to not only genomic data, but also other layers of the omics profiling such as the transcriptome, proteome, and the epigenome.

I have some minor comments to improve the text and optional suggestions that could be implemented as part of this work (if time permits) or in future versions of the software.

- Probably due to the word count limits of the short report format, the text was sometimes too compactly written. A little more explanation could help in certain parts. For instance, a summary of the prediction tasks that have been studied could be added before starting the results section.

In the revised manuscript, we chose to not summarize the prediction tasks before the results as the details felt out of place in terms of the narrative of this short report. We did elect to elaborate on the specifics of each prediction task as their experiments are brought up, which should improve the overall clarity.

- Again probably due to the word limit, a discussion of future directions is missing. What other tasks can this method be used on? Can one use these data augmentations to predict for instance the effect of genomic changes on the target gene expression levels (as in GTEX)? It would be nice to see a few examples discussed in the discussion sections to see how else this tool could be used.

In the revised manuscript, we include additional information on the future directions, focusing mainly on how the improved genetic variation can help to learn variant effects (including eQTLs).

- The authors have demonstrated the generalization power of their training procedure on various tasks, but all of them are within-dataset context. It would be an open question how the model would generalize in a cross-dataset context where the distributions could be more shifted. Possibly a second fine-tuning to the external dataset could solve this issue. Maybe the authors could comment on this in the discussion section.

Thank you for your comment. We appreciate your suggestion about cross-dataset generalization as an interesting task. We opted to focus mainly on in-distribution generalization, but we did show improved generalization on zero-shot predictions on the CAGI5 dataset, which is a different experimental modality. We believe that fine-tuning on this dataset could further improve performance, as shown by Enformer. In the revised manuscript, we have included a more detailed discussion of the generalization capabilities of our models and their limitations. Thank you again for valuable feedback.

- Data augmentation brings up the idea of using contrastive learning approaches using for instance contrastive/triplet loss, which can also be used in a supervised setting. I wonder if the authors considered following such an approach and if it makes any difference.

The data augmentations were originally motivated to enable pretraining of contrastive learning approaches, including Barlow Twins and DeepInfoMax. These methods require different views of the data, and we created augmentations to serve this purpose. While we found that contrastive methods did significantly better, we found that most of the gains came from just the augmentations. In the revised manuscript, we briefly discuss how EvoAug can be used for contrastive learning.

- The authors apply two different sets of hyperparameter optimization: one for learning the neural network model training parameters and one for learning the data augmentation parameters (such as mutation rate or length and combinations of different kinds of augmentations). Hyperparameters are randomly generated on a grid and best performing parameters are selected. This sounds like a good use case where a population based training could be used to detect optimum hyperparameters concurrently for both the augmentation parameters and the model training parameters. In such a setting, the algorithm would actually evolve a set of solutions on the go. As the intermediate solutions that perform well are not discarded but exploited for the subsequent solutions while at the same time being mutated, converging on the best fitting hyperparameter setup could be more efficient for population based training compared to random grid search. This can be done using tools such as Ray Tune that can easily be connected to Pytorch code.

We appreciate reviewer 3 for providing such practical feedback. Using Ray Tune for hyperparameter tuning is an excellent idea, which we will definitely consider in our future work. We have acknowledged this limitation and this potential solution in the revised manuscript. Thank you again for bringing this to our attention and guiding us towards a more efficient approach to hyperparameter tuning.

**2^nd^ round**

**Reviewer 1**

The authors have addressed my concerns. This is a nice paper that will be of interest to the community.

**Reviewer 3**

The authors have addressed the points I raised.
